# Risk factors of neurovascular ageing in women

**DOI:** 10.1111/jne.12777

**Published:** 2019-09-03

**Authors:** Virginia M. Miller, Muthuvel Jayachandran, Jill N. Barnes, Michelle M. Mielke, Kejal Kantarci, Walter A. Rocca

**Affiliations:** ^1^ Departments of Surgery and Physiology and Biomedical Engineering Mayo Clinic Rochester MN USA; ^2^ Department of Physiology and Biomedical Engineering Mayo Clinic Rochester MN USA; ^3^ Division of Nephrology and Hematology Research Department of Internal Medicine Mayo Clinic Rochester MN USA; ^4^ Department of Kinesiology University of Wisconsin‐Madison Madison WI USA; ^5^ Division of Epidemiology Department of Health Sciences Research and Department of Neurology Mayo Clinic Rochester MN USA; ^6^ Department of Radiology Mayo Clinic Rochester MN USA

**Keywords:** aortic blood pressure, brain volume, cognition, oestradiol, menopause, pregnancy, white matter hyperintensities

## Abstract

Biological sex and changes in sex hormones throughout life influence all aspects of health and disease. In women, changes in sex hormonal status reflect ovarian function, pregnancy and the use of exogenous hormonal treatments. Longitudinal data from defined cohorts of women will help to identify mechanisms by which the hormonal milieu contributes to cerebrovascular ageing, brain structure and ultimately cognition. This review summarises the phenotypes of three cohorts of women identified through the medical records‐linkage system of the Rochester Epidemiology Project and the Mayo Clinic Specialized Center of Research Excellence (SCORE) on Sex Differences: (i) menopausal women with histories of normotensive or hypertensive pregnancies; (ii) women who had bilateral oophorectomy ≤45 years of age; and (iii) women who experienced natural menopause and used menopausal hormone treatments for 4 years. Data from these cohorts will influence the design of follow‐up studies concerning how sex hormonal status affects neurovascular ageing in women.

## 1 INTRODUCTION

Biological sex and changes in sex hormones across the lifespan affect all aspects of health and disease. Historically, reviews of sex and gender on brain pathology and cognitive decline have been broad.^1^, ^2^, ^3^, ^4^, ^5^ Few reviews have focused on the effect of hormonal changes on ageing‐related pathophysiology because such studies require long‐term follow‐up and continuous funding across several generations of investigators. Longitudinal data from defined cohorts of women will help to identify mechanisms by which changes in the hormonal milieu contribute to cerebrovascular ageing, brain structure and ultimately cognition. This review summarises the phenotypes of three cohorts of women identified through the medical records‐linkage system of the Rochester Epidemiology Project (REP) and the Mayo Clinic Specialized Center of Research Excellence (SCORE) on Sex Differences: (i) menopausal women with histories of normotensive or hypertensive pregnancies; (ii) women who had bilateral oophorectomy ≤45 years of age; and (iii) women who experienced natural menopause and used menopausal hormone treatments for 4 years.^6^, ^7^, ^8^, ^9^ Cardiovascular risk factors, cardiovascular structure, cerebrovascular reactivity, brain structure and cognition were examined in each of these cohorts. These data provide a foundation for establishing long‐term follow‐up studies directed toward understanding how hormonal changes affect neurovascular ageing in women.

## 2 PREGNANCY HISTORY

**Figure 1:**
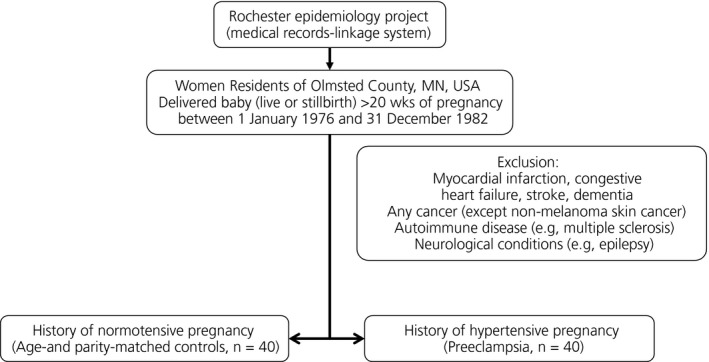
Flow chart characterising the cohorts to examine the long‐term effects of pregnancy on cardiovascular risk factors and cognition in middle‐aged women

The REP medical records‐linkage system was used to identify age and parity matched women living within a 120 mile radius of Rochester, MN who gave birth from 1976 through 1982 with (n = 40) and without (n = 40) a history of pre‐eclampsia.^10^ A history of pre‐eclampsia was determined through medical record abstraction and was defined as new onset, sustained hypertension (systolic blood pressure [SBP] >140 or diastolic blood pressure [DBP] >90 mm Hg) and/or the use of an anti‐hypertensive treatment after 20 weeks of gestation, in combination with new or worsening proteinuria (>300 mg day^‐1^) and/or other features of severe disease (magnesium sulphate administration, elevated liver enzymes, thrombocytopaenia or acute renal failure). Because the primary focus of this study was to understand the intravascular cellular mechanisms that place women with histories of pre‐eclampsia at higher risk for future cardiovascular disease, women who had a medical‐record confirmed clinical diagnosis of myocardial infarction, congestive heart failure, stroke, dementia, any cancer (with the exception of non‐melanoma skin cancer), autoimmune disease (eg, multiple sclerosis, lupus) and neurological conditions (eg, epilepsy) (Figure 1) were excluded. In addition to collection of blood samples for standard assessment of cardiovascular risk factors, women eligible for the study underwent computed tomography to asses coronary artery calcification (CAC),^10^ ultrasound imaging to assess carotid intima medial thickness (CIMT),^11^ magnetic resonance imaging to assess brain volumes and white matter hyperintensities,^12^ cerebrovascular reactivity testing,^13^ cognitive testing,^14^ and evaluation of circulating cell‐derived microvesicles.^15^, ^16^, ^17^

The median age of women at the time of their first pregnancy was 24 years, and women were approximately 60 years of age at the time of their study visit (Table 1). Of the conventional cardiovascular risk factors, only body mass index, waist circumferences and serum insulin levels were greater in women with a history of pre‐eclampsia compared to women with a history of normotensive pregnancy. Although there were no differences in peripheral blood pressure between the groups at the study visit, women with a history of pre‐eclampsia were more likely to have a diagnosis of hypertension and to use antihypertensive medications than women with a history of normotensive pregnancy (Table 1).

**Table 1:** Characteristics of women from the pregnancy history cohort

Characteristic^a^	Normotensive (n = 40)	Pre‐eclampsia (n = 40)	*P* value
Age at study consent	59.6 (56.2, 62.5)	59.2 (56.3, 62.5)	0.81
Age at first live birth	24.0 (22.3, 26.3)	24.5 (21.7, 25.8)	0.93
Clinical parameters			
Body mass index (kg m^‐2^)	25.3 (23.1, 32.0)	29.8 (25.9, 33.7)	**0.02**
Waist circumference (cm)	85.3 (79.3, 99.6)	98.0 (88.3, 104.0)	**0.009**
Systolic blood pressure (mm Hg)	128.7 (116.5, 145.7)	131.7 (119.7, 140.2)	0.61
Diastolic blood pressure (mm Hg)	75.2 (69.7, 84.0)	79.7 (69.3, 83.3)	0.37
Hypertension, chart‐abstracted (n)	8 (20%)	24 (60%)	**<0.001**
Antihypertensive meds, chart‐abstracted	5 (13%)	23 (58%)	**<0.001**
Insulin (μIU mL^‐1^)	4.6 (3.3‐6.0)	7.1 (4.7‐14.8)	**<0.001**

Note

Modified with permisson from White et al.^10^

Bold numbering emphasizes statistical significance.

a Data are shown as median (25th to 75th quartile) for most characteristics; as number of individuals (%) for hypertension and antihypertensive medications; and as median (range) for insulin.

Both CAC and CIMT were greater in women with a history of pre‐eclampsia compared to women with a history of normotensive pregnancy.^10^, ^11^ Distinct sets of circulating cell‐derived microvesicles were associated with each of the two anatomical changes. CAC was associated with microvesicles carrying the antigen for tissue factor and with microvescicles derived from stem cells and adipocytes.^16^ CIMT was associated with the number of platelets, specific markers of platelet activation and microvesicles derived from platelet or platelet‐monocyte interactions.^18^ Although women with a history of pre‐eclampsia had a different metabolic phenotype based on body mass index, waist circumference and insulin sensitivity, their sympathetic metaboreflex to a physical stress were similar to women who had a history of normotensive pregnancy.^19^ These results suggest that there may be different mechanisms leading to progression of CIMT compared to CAC, and that these processes may be distinct from those affecting autonomic reflex to metabolic challenge. Although there are several large observational and randomised studies that investigated cardiovascular risk factors associated with the development of CIMT and CAC in women, none have taken into consideration pregnancy history.^20^, ^21^, ^22^, ^23^, ^24^ A simple, validated questionnaire is available to obtain pregnancy history.^25^ Use of this questionnaire may help to identify women who are at risk for targeted early intervention to control conventional, manageable risk factors such as hypertension, obesity/diabetes and lifestyle (ie, smoking, diet, activity).

**Figure 2:**
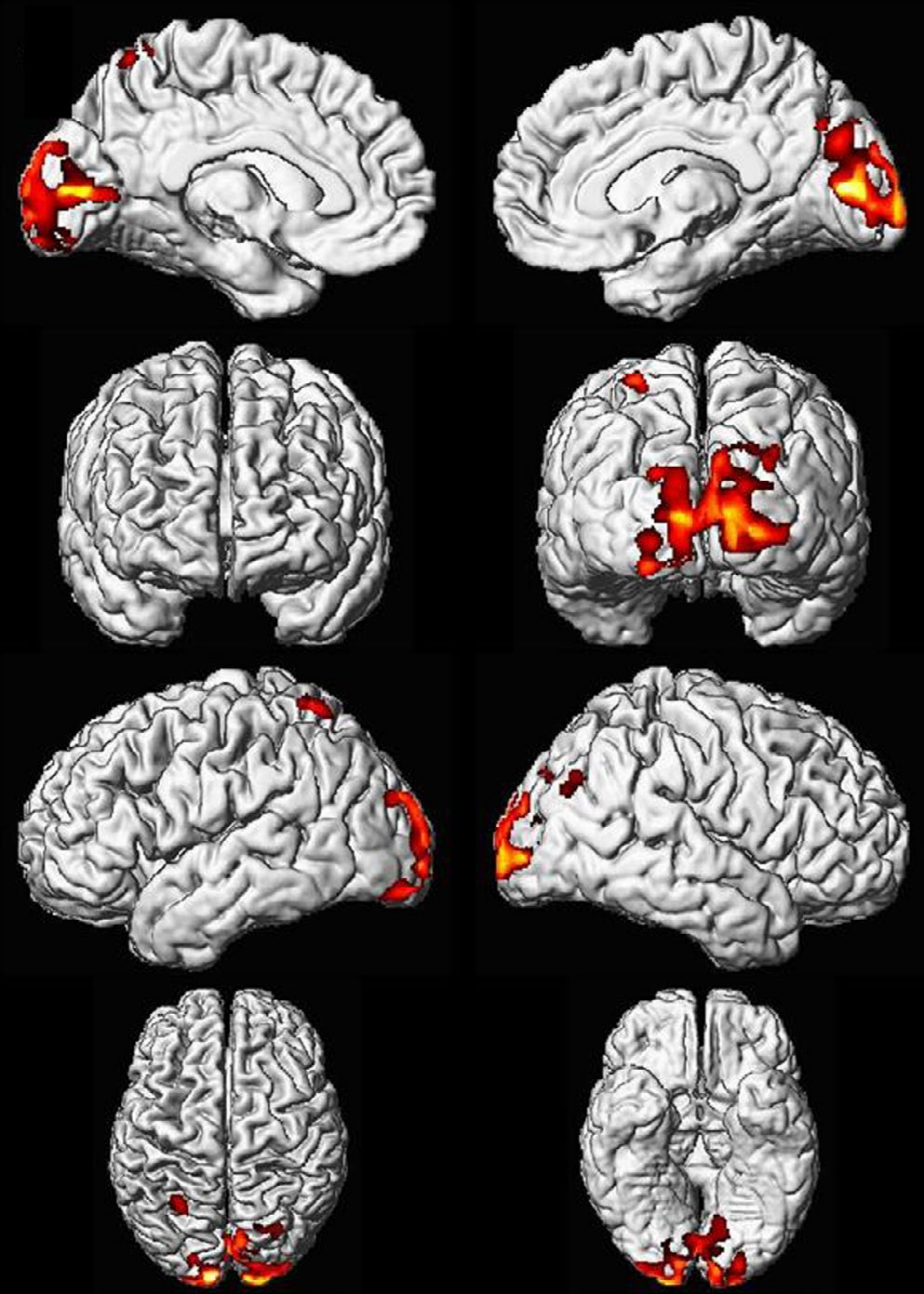
Voxel‐based analysis from magnetic resonance imaging of brains of women with a history of pre‐eclampsia. The red areas are voxels that showed a lower cortical volume in women with a history of pre‐eclampsia and current hypertension compared to women with a history of pre‐eclampsia and no current hypertension (*P* < 0.001). Modified with permission from Raman et al^12^

To investigate potential effects of a history of pre‐eclampsia on blood flow regulation in the brain, middle cerebral artery blood velocity (MCAv) was measured by ultrasound prior to and during stepped hypercapnia. Changes in MCAv were lower in women with a history of pre‐eclampsia at each level of hypercapnia compared to women with a history of normotensive pregnancy, suggesting impaired cerebrovascular reactivity.^13^ Women with a history of pre‐eclampsia, especially those who had current hypertension (Figure 2), showed atrophy in the occipital lobes of the brain and deficits on visual‐spatial cognitive tests.^12^, ^14^ Collectively, these data confirm the results of other studies^26^ showing that women who have had a history of a pre‐eclamptic pregnancy are at risk for accelerated cardiovascular disease, brain atrophy and cognitive impairments, and that these effects can occur in mid‐life. Thus, this cohort provides a unique opportunity for additional follow‐up of neurovascular ageing in women with defined pregnancy histories. These data also confirm the long‐standing association of hypertension with risk of brain structural changes and cognitive decline in women and men,^27^, ^28^, ^29^ and emphasise the need to monitor women with histories of pre‐eclampsia regularly after their pregnancy to treat hypertension at its earliest stage. Changes in recent guidelines in the USA for defining hypertension will help to reduce the adverse effects of hypertension on cerebrovascular function in women by having treatments initiated earlier at lower values of systolic pressure.^30^, ^31^, ^32^

## 3 BILATERAL OOPHORECTOMY

**Figure 3:**
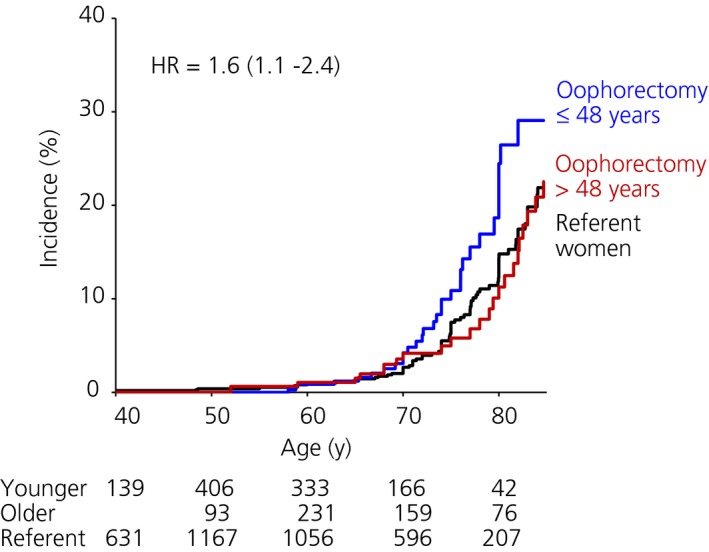
Cumulative incidence of cognitive impairment or dementia in women with bilateral oophorectomy at age ≤48 years or at age >48 years, as well as in referent women. HR, hazard ratio. Modified with permission from Rocca et al^12^

Bilateral oophorectomy abruptly reduces 17β‐oestradiol and all ovarian hormones, including progesterone, testosterone and androstenedione. With the loss of these ovarian hormones, pituitary hormones are increased (ie, follicle‐stimulating hormone). Historically, in animal studies, bilateral ovariectomy has been used as a surrogate for menopause to study the effects of the loss of reproductive hormones, and to determine targeted effects of hormonal treatments, mostly oestrogen, on specific physiological or anatomical outcomes. These studies are not representative of natural menopause with a more gradual decline in hormones. Indeed, they have provided a vast amount of data that can help understand how the abrupt loss of ovarian hormones might affect women who undergo bilateral oophorectomy before the age of natural menopause. Unfortunately, few studies have used an integrated approach to evaluate the long‐term consequences of bilateral oophorectomy in women. To begin to fill this gap in the medical literature, the REP medical records‐linkage system was used to identify women from 1950 to 1987 who underwent bilateral oophorectomy at ≤48 years of age and after 48 years of age to assess long‐term consequences of the procedure in some women up to the age of 80 years. The risk of cognitive impairment and dementia was greater in women who underwent bilateral oophorectomy prior to natural menopause and at age ≤48 years compared to referent women (Figure 3). This risk remained increased after adjustments for education and indication for the oophorectomy.^33^ This fundamental observation has been confirmed by several subsequent studies of other cohorts.^34^, ^35^, ^36^, ^37^

Our initial findings were confirmed in a replicative study of a more contemporary cohort of women from 1988 through 2007, comprising women with bilateral oophorectomy ≤45 years of age and a cohort of referent women. We investigated the effects of bilateral oophorectomy on the incidence of multi‐morbidities and accelerated ageing. At an average of 14 years after the incident event (oophorectomy), women who had the procedure at ≤45 years of age experienced an increase risk of 18 chronic conditions compared to referent women (ie, those age‐matched women had not undergone bilateral oophorectomy).^9^, ^38^ These data are consistent with an overall increase in all cause and disease‐specific mortality observed in the large Breast Cancer Detection Demonstration Project of women who had undergone bilateral oophorectomy.^39^

The 18 chronic conditions that were significantly increased following bilateral oophorectomy included depression, thus suggesting the impact of the abrupt loss of ovarian hormones on neuronal function.^38^, ^40^ Consistent with these clinical findings, women who had bilateral oophorectomy ≤45 years of age had neurodegenerative changes in the medial temporal lobe, with smaller amygdala volumes, thinner para‐hippocampal‐entorhinal cortices and lower white matter fractional anisotropy values in the entorhinal cortex compared to referent women.^34^ These changes may place these women at risk for future cognitive impairment. Although the effects of menopausal hormone treatments on brain structure and cognition in women with bilateral oophorectomy remain to be determined, the collective data suggest that women of average risk of cancer should not use bilateral oophorectomy for a generic prophylaxis of ovarian cancer.

## 4 NATURAL MENOPAUSE AND EXOGENOUS HORMONAL TREATMENT

By contrast to women who experience the abrupt hormonal drop following bilateral oophorectomy, women who undergo natural menopause experience fluctuations in ovarian hormones for years prior to the cessation of menses.^41^ These fluctuations are followed by declines in oestrogen and increases in follicle‐stimulating hormone. Although several large scale observational and randomised trials have examined the impact of menopausal hormone changes and treatments on measures of cognition, the studies were limited by the inclusion of women with a broad range of ages and women with pre‐existing cardiovascular disease, as well as women who had undergone natural menopause and those who had either unilateral or bilateral oophorectomy.^42^, ^43^, ^44^, ^45^, ^46^, ^47^, ^48^ By contrast, the women enrolled in the Kronos Early Estrogen Prevention Study (KEEPS) provided an opportunity to study changes in cognition in a defined group of women who had undergone natural menopause and in whom cardiovascular risk factors, including the presence of CAC, were low (Table 2).^6^, ^49^

**Table 2:** Characteristics of women enrolled in the Kronos Early Estrogen Prevention Study^a^

Characteristic	N	Mean ± SD
Age (years)	728	52.7 ± 2.6
Time past menopause (years)	722	1.2 ± 0.6
Body mass index (kg m^‐2^)	722	26.2 ± 4.3
Systolic blood pressure (mm Hg)	728	118.6 ± 15.1
High‐density lipoprotein cholesterol (mg dL^‐1^)	728	65 ± 11.o
Low‐density lipoprotein cholesterol (mg dL^‐1^)	728	129 ± 29.0
Fasting glucose (mg dL^‐1^)	728	89.1 ± 9.9
Follicle stimulating hormone (IU L^‐1^)	364	90.36 ± 36.0
17β oestradiol (pg mL^‐1^)	364	16.9 ± 30.0
Never smokers	580	78%

a Modified with permission from Miller et al^12^ and Harman et al^12^, as well as unpublished observations for follicle‐stimulating hormone.

KEEPS was a double‐blind, placebo‐controlled trial enrolling women between the ages of 42 and 58 years who were between 6 months and 3 years since their last menstrual period aiming to study the effects of two different menopausal hormone treatments on progression of cardiovascular disease measured by changes in CIMT. Women in KEEPS were randomised to one of three treatments: 0.45 mg day^–1^ oral conjugated equine oestrogen (o‐CEE); 50 μg day^–1^ transdermal 17β‐oestradiol (t‐E_2_) or placebo pills and patches. Women in the active treatment groups also received oral 200 mg day^–1^ micronised progesterone for the first 12 days of each month to protect the endometrium. These formulations of treatments were different from those used in the Women's Health Initiative Studies (ie, a lower dose of conjugated equine oestrogen and in women with a uterus, the natural progesterone instead of the synthetic progestogen medroxyprogesterone acetate.)^44^ Women enrolled in KEEPS had the opportunity to participate in a study to evaluate cognition and mood,^50^ and a subset had the opportunity to enroll in a study of brain imaging.^15^, ^51^

Prior to randomisation, scores on the cognitive tests were within the normative range. However, there was a negative correlation between scores on tests for cognition/working memory and systolic blood pressure (*t* = −2.93; *P* = 0.004), confirming the importance of controlling systolic blood pressure during midlife. No correlation was found with any other of the conventional cardiovascular risk factors.^52^ Prior to randomisation, all women had similar amounts of white matter hyperintensities on magnetic resonance imaging of their brains.^15^ The volume of white matter hyperintensities increased over the 4 years of the trial, although it was not modified by menopausal hormone treatment (Table 3)^51^ and was associated with thrombogenicity of the blood as measured by thrombogenic microvesicles in the blood at the time of randomisation.^15^ These results suggest that factors affecting the development of white matter hyperintensities, such as central aortic blood pressure^53^ and thrombogenic microvesicles, may precede changes measurable with imaging.

**Table 3:** Changes in brain structures prior to, during, and following randomisation to menopausal hormone treatments in the Kronos Early Estrogen Prevention Study (KEEPS)^a^

Characteristic	Baseline	During treatment	Following treatment	Reference
Cognitive test scores	Normative range; no differences among group assignments	Normative range; no differences among group assignments	Normative range; no difference among prior treatment group assignments	54, 55
Cortical volume	No differences among group assignments		Cortical preservation t‐E_2_ > PLO	51, 55
Ventricular volumes	No differences among group assignments	o‐CEE > PLO	No differences among prior treatment groups	51, 55
White matter hyperintensities	o‐CEE > t‐E_2_, PLO	Increased in all groups; no treatment effect	Increased in all prior treatment groups; o‐CEE > PLO	51, 55
β‐amyloid			t‐E_2_ < o‐CEE, PLO specifically in *APOE* ε4 carriers	56

Abbreviations: o‐CEE, oral conjugated equine oestrogen, t‐E_2_, transdermal 17β‐oestradiol; PLO, placebo.

a Baseline is defined as prior to randomisation; during treatment is at the 48‐month visit; following treatment is 3 years after cessation of the trial.

The association between increases in blood pressure and development of white matter hyperintensities observed in KEEPS is consistent with what has been observed in women with a history of hypertensive pregnancy disorders, as well as in other cohorts of men and women as discussed above. The consistency of this association between blood pressure and brain structural and functional changes should be embedded into the rationale for the treatment of hypertension. Additional studies are needed to better understand the relationship between the cumulative burden of hypertension and the rate of change in brain structures and deficits in cognitive performance, as well as how those relationships might be impacted by the initiation of anti‐hypertensive treatments.

Although the hormonal treatments used in KEEPS did not significantly increase in white matter hyperintensities, the ventricular volume increased and cortical volume decreased more in the o‐CEE group than in the t‐E_2_ or placebo groups,^51^ suggesting that the hormonal formulations have differential effects in the brain and that the threshold for these effects may not be the same for all cells types. Despite these changes in brain structure, there were no differences in cognitive performance among the groups after 4 years of treatment. The participants were relatively young at the time of the study, and structural changes may occur at an earlier age than cognitive changes.^51^, ^54^

**Figure 4:**
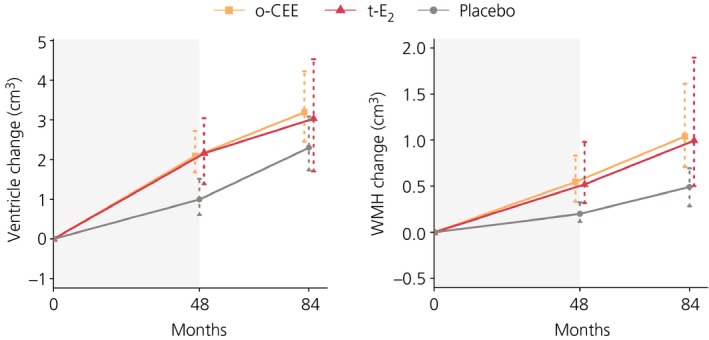
Longitudinal change in ventricular volumes and white matter hyperintensities (WMH) in women who had been randomised to placebo, oral conjugated equine oestrogen (o‐CEE) or transdermal 17β‐oestradiol (t‐E_2_) for 4 years. Data are shown as the mean ± SD prior to randomisation (0 months), at the conclusion of treatment (48 months), and 3 years after cessation of treatment (84 months). Modified with permission from Kantarci et al^12^

A subset of women (n = 75) underwent brain imaging and cognitive testing 3 years after the cessation of the KEEPS study. In these women, increases in ventricular volumes did not differ based on prior treatment assignment. White matter hyperintensities continued to increase in all groups; however, women previously assigned to o‐CEE had greater increases compared to the placebo group. Decreases in the volume of the dorsolateral prefrontal cortex were less in women previously assigned to the t‐E_2_ group compared to placebo.^55^ Accumulation of β‐amyloid as measured by uptake of Pittsburgh compound B also was lower in the t‐E_2_ group. In particular, accumulation of β‐amyloid was lower in women who were carriers of the APOE4 genotype and were assigned to t‐E_2_ (Figure 4).^55^, ^56^ These observations suggest that there may be a gene‐hormone interaction involving the circulating concentrations of oestrogen and oestrogen metabolites (o‐CEE vs t‐E_2_) that may reduce accumulation of β‐amyloid in APOE4 carriers.

Although there were measurable changes and differences among prior treatment groups in regard to brain volume, white matter hyperintensities and accumulation of β‐amyloid, there were no significant differences in cognitive performance among groups. Long‐term follow‐up of women from KEEPS provides a unique opportunity to determine whether these structural changes will affect cognition as women age (Table 3). Unfortunately, pregnancy history was not captured in the initial intake forms for KEEPS. However, a questionnaire will be administered in conjunction with future follow‐up of the entire KEEPS cohort. These new data will allow further assessment of how hypertension, pregnancy and hormonal treatments are related to neurovascular ageing.

## 5 SUMMARY

Studies of how hormonal changes contribute to risk factors that may slow or accelerate the natural ageing processes and associated disease processes require continuous funding, perhaps across several generations of investigators. Although sustaining funding for long‐term follow‐up studies is an ongoing issue, our group has leveraged the REP medical records‐linkage system and the Mayo Clinic SCORE on sex differences to identify three cohorts of middle‐aged women. These cohorts provide an opportunity for long‐term follow‐up to investigate how vascular changes during pregnancy and changes in sex hormones following surgical or natural menopause affect ageing processes, neurovascular structure and function, and cognition in women. Data from these cohorts should inform the design and direction of future larger studies of the effects of sex, hormones and ageing on neurovascular function. In particular, future studies should collect data on pregnancy history, ovarian status and hormonal status. Although this review has focused on the abrupt loss of ovarian function as a result of bilateral oophorectomy, other ovarian conditions causing premature ovarian insufficiency, such as exercise‐induced amenorrhoea, eating disorder induced irregular menses and polycystic ovarian syndrome, provide potential cohorts of interest. Hormone treatment type, dose and mode of delivery should be considered, as well as more complete studies of duration of hypertension or its treatment, in relation to brain imaging and functional testing. Although findings from defined cohorts in a particular population are often criticised because they may not be applicable to the general population, they provide information for targeting specific approaches that can lead to a better understanding of the underlying pathophysiological processes associated with ageing.

## CONFLICT OF INTERESTS

The authors declare that they have no conflicts of interest.

## Data Availability

Data sharing is not applicable to this article because no new data were created or analysed in the present study.
